# Diagnostic Value of FDG PET/MRI in Females With Pelvic Malignancy—A Systematic Review of the Literature

**DOI:** 10.3389/fonc.2020.519440

**Published:** 2020-09-29

**Authors:** Nghi Co Nguyen, Sushil Beriwal, Chan-Hong Moon, Nicholas D'Ardenne, James M. Mountz, Alessandro Furlan, Ashok Muthukrishnan, Balasubramanya Rangaswamy

**Affiliations:** ^1^Department of Radiology, University of Pittsburgh, Pittsburgh, PA, United States; ^2^Department of Radiation Oncology, School of Medicine, University of Pittsburgh, Pittsburgh, PA, United States

**Keywords:** FDG PET, PET/CT, PET/MRI, female', pelvic malignancy

## Abstract

Hybrid imaging with F-18 fludeoxyglucose positron emission tomography/magnetic resonance imaging (FDG PET/MRI) has increasing clinical applications supplementing conventional ultrasound, CT, and MRI imaging as well as hybrid PET/CT imaging in assessing cervical, endometrial, and ovarian cancer. This article summarizes the existing literature and discusses the emerging role of hybrid PET/MRI in gynecologic malignancies. Thus, far, the published literature on the applications of FDG PET/MRI shows that it can have a significant impact on patient management by improving the staging of the cancers compared with PET/CT, influencing clinical decision and treatment strategy. For disease restaging, current literature indicates that PET/MRI performs equivalently to PET/CT. There appears to be a mild-moderate inverse correlation between standard-uptake-value (SUV) and apparent-diffusion-coefficient (ADC) values, which could be used to predict tumor grading and risk stratification. It remains to be seen as to whether multi-parametric PET/MRI imaging could prove valuable for prognostication and outcome. PET/MRI provides the opportunity for reduced radiation exposure, which is particularly relevant for a young female in need of multiple scans for treatment monitoring and follow-up. Fast acquisition protocols and optimized methods for attenuation correction are still evolving. Major limitations of PET/MRI remains such as suboptimal detection of small pulmonary nodules and lack of utility for radiation treatment planning, which pose an impediment in making PET/MRI a viable one-stop-shop imaging option to compete with PET/CT.

## Introduction

Gynecologic malignancies are common causes of morbidity and mortality in women (1). The International Federation of Gynecology and Obstetrics (FIGO) system is used for staging of most pelvic malignancies in women (2), which is based on the physical exam and a few other procedures such as colposcopy, conization of the cervix, cystoscopy, and rectosigmoidoscopy. A major limitation of FIGO staging is that it lacks consideration of locoregional nodal evaluation so advanced imaging modalities (computed tomography, CT; magnetic resonance imaging, MRI; and positron-emission tomography, PET) are often necessary. In this regard, PET/CT with F-18 fludeoxyglucose (FDG) is a valuable modality for initial staging and restaging of pelvic malignancies in women (3–7). Contrast-enhanced (ce) MRI is an established imaging modality that has numerous clinical applications due to its superb soft tissue contrast and lack of ionizing radiation, and the ability to assess cellular density by diffusion-weighted imaging (DWI) and tissue perfusion by dynamic contrast-enhanced (DCE) (6, 8, 9). MRI also has the potential to complement the metabolic imaging provided by PET. Therefore, the combination of PET and MRI in an integrated (hybrid) PET/MRI system promises to have a positive impact on disease diagnosis, staging, and restaging (10). One unique advantage of PET/MRI is a significant reduction of radiation exposure which can be as much as 45% in young patient populations compared with PET/CT (11). The goal of this article is to summarize the existing literature and discuss the emerging role of hybrid PET/MRI in gynecologic malignancies.

## Review of Literature

For the review of the literature, we performed a PubMed search to find relevant articles about the diagnostic value of PET/CT and PET/MRI in women with pelvic malignancies. The following keywords were used: PET/CT; CT/PET; PET/MR; PET/MRI; MR/PET; MRI/PET; carcinoma; cancer; pelvis; female. PubMed was searched from January 1993 through June 2019, limited to the English language. Studies with FDG PET/CT and FDG PET/MRI (fused/co-registered or integrated) in women with pelvic malignancies were reviewed. Studies with separate evaluations of PET and MRI were excluded. Case reports, case series, review articles, letters, comments, preclinical studies, and animal studies were excluded.

We have identified 26 studies consisting of a total of 801 subjects that reported on the diagnostic value of fused or integrated PET/MRI for the clinical management of female pelvic malignancies, Table 1. Of these, 17 studies were based on an integrated PET/MRI system (12–18, 26–29, 31–34, 36, 37), and nine studies applied co-registered (fused) PET/MRI data (19–25, 30, 35). A total of 15 studies with 413 subjects were for staging (12–25, 27, 36); six studies with 187 subjects for restaging (31–35, 37); and six studies of 175 subjects of mixed staging and restaging (26–30, 36). Eighteen studies with 462 patients reported on the diagnostic potential of MRI with DWI (MR/DWI). One study evaluated the prognostic potential of PET/MRI (14). Two studies were designed to validate a fast whole-body PET/MRI scanning (32, 34).

**Table 1:** Summary of PET/MRI (fused, integrated) literature on pelvic malignancy in women.

**ID**	**References**	**Study Design**	**IND**	**Sample size [Primary]**	**SUV & ADC**	**Aim/Results/Conclusions**
1	Schwartz et al. (12)	P	Staging	18 [C-11; EU-7]	No	To compare integrated FDG PET/MRI with PET/CT. PET/neMRI detects all primaries c/w PET/ neCT; PET/neMRI detects parametrial invasion & one patient had invasion of the bladder, not detected on PET/neCT.
2	Meyer et al. (13)	R	Staging	18 [C-18]	Yes	To correlate ADC with FDG PET derived from integrated PET/MRI. SUVmax or SUVmean do not correlate with ADC. SUV-based volumes correlate inversely with ADC.
3	Floberg et al. (14)	R	Staging	17 [C-17]	Yes	To investigate spatial relationship between SUV & ADC and correlate with clinical outcome. Consistent inverse correlation between SUV & ADC was noted in SCCA and poorly differentiated tumors, which may have prognostic significance in terms of disease-free survival.
4	Surov et al. (15)	P	Staging	21 [EU-21]	Yes	To correlate between SUV & ADC in predicting histopathological features. No significant change in SUV & DWI noted between different tumor grades (G1-3). However, combined SUV & ADC provide the greatest correlation with Ki-67 which could be valuable to estimate the cell proliferation of the primary.
5	Grueneisen et al. (16)	P	Staging	27 [C-27]	Yes	To provide initial results on integrated PET/MRI. PET/ceMRI shows high potential for the assessment of primary and nodal metastases, with 93% diagn accuracy for nodal disease. SUV & ADC reveal a significant correlation with pathological grade and tumor size.
6	Shih et al. (17)	P	Staging	36 [EU-36]	Yes	To correlate between SUV & ADC. SUVmax & ADCmin at integrated PET/enMR are inversely correlated and associated with pathologic prognostic factors. Combined parameters provide the greatest correlation with pathologic biomarkers.
7	Sun et al. (18)	P	Staging	35 [C-35]	Yes	To correlate tumor volumes between PET, MRI and DWI. Strong volume concordance is observed between PET, and MRI & DWI. Cut-off at 35% or 40% SUVmax is recommended for SUV-based tumor volume rendering.
8	Stecco et al. (19)	R	Staging	27 [C-13; EU-14]	No	To compare PET/CT with fused PET/MRI- DWI, and MRI-DWI. For pelvic staging, AUC is 0.93 for ceMRI-DWI, 0.93 for PET/neCT, and 0.96 for PET/ceMRI-DWI. Although not statistically significant (*p* = 0.055), fused PET/ceMRI-DWI might provide higher diagn accuracy c/w PET/neCT.
9	Pinker et al. (20)	P	Staging	16 [C-16]	Yes	To correlate between fused FDG PET, FMISO PET and MRI-DWI. All tumors display restricted diffusivity, high FDG avidity, and FMISO avidity. Weak correlations between ceMRI and PET parameters indicate that each individual parameter provides independent information on tumor pathophysiology.
10	Olsen et al. (21)	R	Staging	20 [C-20]	Yes	To compare PET/CT with MRI. Concordance between PET and DWI is observed. Tumor sub-volumes with increased FDG avidity also shows greater cell density by DWI.
11	Kitajima et al. (22)	R	Staging	30 [C-30]	No	To compare fused PET/CT with MRI. Diagn accuracy of fused PET/ceMRI and ceMRI (83.3% each) for T staging is higher c/w PET/ceCT (53.3%), *p* = 0.008. Accuracy for nodal metastasis is 90.0% for fused PET/ceMRI and PET/ceCT & 86.7% for ceMRI.
12	Kitajima et al. (23)	R	Staging	35 [EU-35]	No	To compare fused PET/CT with MRI. Fused PET/ceMRI and ceMRI detects 96.7% of primary, whereas PET/ceCT detects 93.3%. Accuracy for T status is 80.0% for PET/ceMRI; ceMRI is more accurate than PET/ceCT. PETce/MRI complements the individual advantages of ceMRI and PET.
13	Anner et al. (24)	R	Staging	27 [C-27]	No	To compare PET/CT and fused PET/MRI. PET/ceCT and fused PET/ceMRI show same sensitivity (64%) for pelvic N-staging. Fused PET/ceMRI does not lead to better results than PET/ceCT.
14	Kim et al. (25)	R	Staging	79 [C-79]	No	To compare PET/CT with fused PET/MRI. For pelvic N-staging, the sensitivity and specificity of PET/neCT and fused PET/ceMRI are 44.1%, 93.9% and 54.2%, 92.7%. Fused PET/ceMRI has added value to PET/neCT for nodal metastasis.
15	Xin et al. (26)	P	Staging/restaging	20 [C-15; EU-3; O-2]	No	To provide initial results on integrated PET/MRI c/w PET/CT. Lesion detection in abdomen/pelvis is similar between PET/neCT and integrated PET/neMRI, although PET/neMRI detects 3 additional foci of early cervical cancer.
16	Grueneisen et al. (27)	P	Staging/restaging	48 [C-22; EU-4; O-9; VV-13]	No	To determine diagn value of DWI in PET/MRI. PET provides greater diagn confidence than DWI (*p* < 0.05). DWI in PET/ceMRI has no diagn benefit for whole-body staging of women with pelvic malignancies.
17	Grueneisen et al. (27)	P	Staging/restaging	19 [C-19]	Yes	To correlate between SUV & ADC. Integrated PET/ceMRI shows high diagn potential. SUVmax and ADCmin reveal a strong inverse correlation in primary and nodal metastases (R = −0.692, < 0.001), not in recurrent cancer.
18	Queiroz et al. (28)	P	Staging/restaging	25 [C-7; EU-5; O-12; VV-1]	No	To compare integrated PET/MRI with PET/CT. Integrated PET/ceMRI is superior to PET/ceCT for primary tumor delineation (*p* < 0.001). No difference was found in detection of regional nodal/abdominal metastases.
19	Brandmaier et al. (29)	P	Staging/restaging	31 [C-31]	Yes	To correlate SUV with ADC. There are significant inverse correlations between SUVmax and ADCmin, with *r* = −0.532 (*p* = 0.05) in primary tumors, *r* = −0.362 (*p* = 0.05) in primary metastasis, and *r* = −0.747 (*p* = 0.002) in recurrent local tumors.
20	Nakajo et al. (30)	R	Staging/restaging	31 [C-25; EU-3; O-3]	No	To compare fused FDG PET/CT with PET/CT. Fused PET/neMRI performs better than PET/neCT for pelvic malignancies (*P* < 0.01).
21	Sawicki et al. (31)	P	Restaging	71 [C-32; EU-7; O-26; VV-6]	No	To compare integrated PET/MRI with MRI alone. PET/ceMRI better detects recurrence than ceMRI (100 vs. 83.6%, *p* < 0.01), particularly for nodal and peritoneal metastases <1 cm.
22	Kirchner et al. (32)	P	Restaging	43 [C-12; EU-4; O-23; VV-4]	No	To implement a fast PET/MRI protocol. Fast PET/ceMRI provides equivalent diagn performance and exam time c/w PET/ceCT. On a lesion-based analysis, the accuracy is 92% for PET/ceCT and 94% for PET/ceMRI.
23	Beiderwellen et al. (33)	P	Restaging	19 [C-6; O-13]	No	To compare integrated PET/MRI with PET/CT. Lesion detection is similar between PET/ceCT (98%) and PET/ceMRI (97%). Diagn confidence is higher with PET/MRI in malignant (*p* < 0.01) and benign lesions (*p* < 0.05).
24	Grueneisen et al. (34)	P	Restaging	24 [C-7; EU-4; O-13]	No	To implement a fast FDG PET/MRI protocol. Fast PET/ceMRI with DWI provides comparably high diagn performance c/w PET/ceCT in restaging. On a lesion-basis, the diagnostic accuracy is 84% for PET/ceCT and 86% for PET/MRI (*p* > 0.05).
25	Grueneisen et al. (34)	P	Restaging	34 [C-18; O-16]	No	To compare integrated PET/MRI with MRI. PET/ceMRI and ceMRI correctly identify 98.9% and 88.8% of malignant lesions. PET/ceMRI provides higher lesion contrast and diagn confidence c/w ceMRI.
26	Kitajima et al. (35)	R	Restaging	30 [EU-21; O-9]	No	To compare fused PET/CT with MRI. On a patient basis, the accuracy for pelvic recurrence/metastasis is 93.3% for fused PET/ceMRI and 86.7% for ceMRI, 86.7% for PET/ceCT & 80.0% for PET/neCT. Fused PET/ceMRI performs significantly better than PET/neCT (*p* = 0.041) only.

*PET indicates FDG PET unless mentioned otherwise. IND, indication; P, prospective; R, retrospective; C, cervical cancer; EU, endometrial/uterine cancer; O, ovarian cancer; VV, vaginal/vulvar cancer; diagn, diagnostic; c/w, compared with; SUV, standard uptake value; DWI, diffusion weighted imaging; ADC, apparent diffusion coefficient; ce, contrast enhanced; ne, nonenhanced*.

In the past 10 years, there has been significant research on the role of PET/MRI in the clinical management of pelvic malignancies in women. A recent meta-analysis in 2017 consisting of seven studies, with a total of 215 subjects for staging and restaging, showed that fused and integrated PET/MRI data provide high diagnostic accuracy in gynecologic malignancies of the pelvis (38). On a per-patient basis, the pooled sensitivity and specificity of FDG PET/MRI were 0.95 (95% CI 0.86 ± 0.99) and 0.95 (95% CI 0.74 ± 1.00). On lesion-based basis, the pooled sensitivity and specificity were 0.89 (95% CI 0.84±0.93) and 0.87 (95%CI 0.74±0.95). The overall area under the curve (AUC) was 0.968 (standard error 0.026).

### Staging

#### Cervical Cancer

Current National Comprehensive Cancer Network (NCCN) guidelines do recommend imaging (CT, PET/CT, and MRI) for stage IB2 or higher, and as an option for stage IB1 or lower (39). The favorable diagnostic accuracy of fused PET/MRI in cervical cancer staging has been demonstrated in various reports (20, 22, 24, 25), with most tumors showing high FDG avidity and enhanced MRI delineation of the primary. In a study with 79 cervical cancer patients, Kim et al. demonstrated that fused PET/MRI had greater sensitivity and specificity compared with PET/CT for N staging (54.2%, 92.7% vs. 44.1%, 93.9%; *p* = 0.026) (25). Grueneisen et al. found that hybrid PET/ceMRI provided correct T-staging in 23 of 27 patients (85%) with cervical cancer (16). Sensitivity, specificity, and diagnostic accuracy for nodal disease were 91, 94, and 93%, respectively. The results of subsequent studies support the high diagnostic potential of hybrid PET/MRI in cervical cancer staging (12, 26–28).

#### Endometrial and Uterine Cancer

NCCN guidelines for endometrial cancer recommend imaging for evaluation of extra-uterine disease, as indicated by clinical workup (40). Thus, in patients with deep myometrial invasion, imaging is commonly used for initial staging. A retrospective study by Kitajama et al. showed that fused FDG-PET/ceMRI yields greater diagnostic performance than PET/ceCT for the evaluation of nodal and distant metastasis in uterine cancer staging (23). Accuracy for T staging was 80.0% for fused PET/MRI, and MRI proved significantly more accurate than PET/ceCT, which had an accuracy of 60.0% (*p* = 0.041). In another retrospective study with 27 patients with endometrial and cervical, the AUC was 0.929 (0.886–0.960) for MRI/DWI, 0.933 (0.891–0.962) for PET/CT, and 0.963 (0.928–0.984) for fused PET/MRI/DWI. However, these values were not statistically significant between hybrid PET/CT and fused PET/MRI/DWI (*p* = 0.055) (19). PET/MRI for tumor staging typically includes a dedicated scan of the pelvis with Gd contrast administration and a scan of the torso.

#### Ovarian Cancer

The role of imaging has also been emphasized in the NCCN guidelines for ovarian cancer (41). MR imaging has been reported to be 95% sensitive and 82% specific for ovarian cancer staging (6). To date, there is a lack of PET/MRI literature (fused or integrated) focusing on ovarian cancer staging. Previous reports include subjects for both staging and restaging, and the sample size was rather small, so the diagnostic value of PET/MRI cannot be adequately assessed (26–28, 30). As an example, Grueneisen et al. found in a study that included nine patients with ovarian cancer that integrated PET/ceMRI had a sensitivity, specificity, and diagnostic accuracy of 92.9, 87.5, and 91.8%, respectively (27). In another study by Queiroz et al. that included 12 ovarian cancer patients, the performance of integrated PET/ceMRI was higher than that of PET/ceCT (*p* < 0.001) (28). No differences were found, however, in the detection of regional nodal or abdominal metastases.

### Restaging

In the most recent meta-analysis comprising of 7 articles with a total of 257 subjects, Zheng et al. showed that FDG PET/MRI yields high diagnostic accuracy in detecting recurrent pelvic malignancies, with pooled sensitivity and specificity of 0.96 and 0.95 (42). The role of PET/MRI in pelvic restaging, however, has only been assessed broadly without focusing on specific tumor entities (16, 31–33, 35, 37, 42). Kitajima et al. showed in a retrospective study of 30 patients (uterine 15; ovarian nine; endometrial six) that fused PET/ceMRI provided better sensitivity for diagnosing local recurrence than PET with nonenhanced CT (PET/neCT) (*p* = 0.041), but it was not better than PET/ceCT or ceMRI (35). The patient-based sensitivity, specificity and accuracy for the detection of pelvic recurrence/metastasis were 91.3, 100, and 93.3% for fused PET/MRI; 78.3, 85.7, and 80.0% for PET/neCT; 82.6, 100, and 86.7% for PET/ceCT; and 82.6, 100 and 86.7% for ceMRI. In a retrospective study by Sawicki et al. with 71 females with suspected recurrence, PET/ceMRI offered greater diagnostic confidence in lesion detection compared with ceMRI (2.7 ± 0.5 vs. 2.4 ± 0.7, *p* < 0.001) as well as diagnostic accuracy (99.2 vs. 79.3%, *p* < 0.001) (31). While PET/ceMRI correctly identified all 181 (100%) malignant lesions, ceMRI correctly identified 135/181 (74.6%) lesions (*p* < 0.001). Also, a considerable number of subcentimeter nodal metastases were FDG avid on PET/MRI but were incorrectly interpreted as benign on ceMRI. The diagnostic contribution of PET scanning was substantiated by the fact that four local recurrences were clearly demonstrated on PET but were only discrete at MRI as well as not associated with diffusion restriction on DWI. The same applied to peritoneal metastases, which were often obscured by adjacent bowel structures or misinterpreted as scar tissue on ceMRI (31). Grueneisen et al. showed in a prospective study of 34 patients (cervical = 18; ovarian = 16) that hybrid PET/ceMRI correctly identified 88 (98.9%) lesions, whereas ceMRI was correct in 79 lesions (88.8%).

### SUV and DWI Correlation

Currently, most functional MRI clinical applications as part of hybrid PET/MRI apply DWI, which informs about the water diffusivity in tissues and providing valuable information on tissue cellularity and membrane integrity. Most reports correlating SUV with apparent diffusion coefficient (ADC) are based on fused or hybrid PET/MRI data, focusing on cervical cancer (13, 14, 16, 18, 20, 21, 29, 36), and only one study each is about endometrial cancer and uterine cancer each (15, 17).

#### Cervical Cancer

In a prospective study of 19 subjects with cervical cancer (staging 10; restaging 9), Grueneisen et al. showed a significant but rather weak inverse correlation between SUVmax and minimum ADC (ADCmin), with R = −0.342, *p* < 0.05 (36). When subdivided into primary and recurrent tumors, primary tumors and associated nodal metastases demonstrated a moderate inverse correlation between SUVmax and ADCmin (R = −0.692, *p* < 0.001). In recurrent lesions, however, there was no significant correlation. In another hybrid PET/MRI study with 31 cervical cancer patients (staging 14, restaging 17), Brandmeier et al. showed an inverse correlation between SUVmax and ADCmin for both primary tumors (r = −0.532, *p* = 0.05) and primary metastases (r = −0.362, *p* = 0.05), as well as recurrent local tumors (r = −0.747, *p* = 0.002) (29). Grueneisen et al. demonstrated in a prospective study with 27 subjects with newly diagnosed cervical cancer, that SUVmax and ADCmin values correlated significantly with pathological grade (well- and moderately vs. poorly differentiated) and tumor size (*p* < 0.05) (16). No significant difference was seen for SUVs between patients with early (stage IB-IIA) or advanced (stage IIB-IVA) tumor stages. In contrast, significantly lower ADCmin values were noted for primary cervical cancers with advanced tumor stages. In addition, SUV or ADC values did not show a significant correlation with tumor histology (squamous cell carcinoma vs. adenocarcinoma) and nodal status.

A significant inverse correlation between SUVmax and mean ADC (ADCmean) was also reported in a recent study by Floberg et al. with 17 newly diagnosed cervical cancer patients (14). Specifically, squamous cell carcinomas (SCCAs) and poorly differentiated tumors consistently showed a significant inverse correlation between voxel SUV and ADC values; but adenocarcinomas and well/moderately differentiated tumors did not. On the other hand, Pinker et al. found only weak correlations between MRI and PET parameters with correlation coefficients ranging from 0.05 to 0.22 in a study with 16 locally advanced cervical cancer patients (20). Also, Meyer et al. showed no statistically significant correlations between SUVmax or SUVmean and ADC parameters in a study with 18 newly diagnosed cervical cancer patients. Still, total lesion glycolysis (TLG) and metabolic tumor volume (MTV) correlated inversely with ADC parameters (13).

#### Uterine and Endometrial Cancer

In a study focusing on uterine cancer, Surov et al. found that the combination of SUV and ADC provided the greatest correlation with the proliferation biomarker Ki-67 (15). Ki-67 correlated significantly with SUVmax (*r* = 0.59, *p* = 0.005) and ADCmin (0.48, *p* = 0.03). SUVmax/ADCmean ratio showed the greatest correlation with (0.75, *p* =0.001). SUVmax correlated well with epithelial area positive for p16 (*r* = 0.71, *p* = 0.001) and stromal area (*r* = −0.71, *p* = 0.001) reflecting metabolically active tumor areas. There were, however, no significant differences in SUV and DWI values between different tumor grades (G1-3) and between T2 and T4 tumors.

In a report by Shih et al. with 36 newly diagnosed endometrial cancer patients, there was a significant inverse correlation between SUVmax and ADCmin (r = −0.53; *P* = 0.001) (17). SUVmax was significantly higher in advanced-stage tumors, deep myometrial invasion, cervical invasion, lymphovascular involvement, and nodal metastasis (P<0.05), but not with tumor grade. ADCmin was lower in higher-grade tumors, advanced stage, and cervical invasion (*P* < 0.05), but not with myometrial invasion, lymphovascular invasion, or nodal metastasis. Most notably, the combined use of SUVmax/ADCmin ratio was associated with all pathologic biomarkers indicating that hybrid PET/MRI may have the potential to provide prognostic information in endometrial cancer. A correlation of SUV and ADC in the context of fused or integrated PET/MRI has not been reported for ovarian cancer to date. Studies above regarding cervical and uterine, as well as endometrial cancers, indicate variable mild to moderate negative correlations between SUV and ADC measures. The high variations in the correlation coefficients may be dependent on the tumor subtypes and may reflect various tumor biologic makeup of the lesions such as hypoxia, and tumor necrosis (43).

### Prognostication

Floberg et al. are the only group to date that reports on the prognostic value of hybrid PET/MRI in gynecologic malignancies in women (14). In a retrospective study of 17 patients with newly diagnosed cervical cancer, they found a significant inverse correlation between SUVmax and ADCmean in SCCAs as well as poorly differentiated tumors. Based on log-rank analysis, the relationship between SUV and ADC was found to be prognostic of disease-free survival (DFS), *p* = 0.026.

### Limitations of PET/MRI

PET/MRI does have its negative attributes. A whole-body PET/ceMRI with a regional scan such as the pelvis or liver takes ~45 min, and a whole-body PET/neMRI with DWI also lasts typically 45 min, which often causes patient discomfort and dissatisfaction. Published reports on fast PET/MRI protocols to reduce the scan time are encouraging, and further validations are required (32, 34). Other disadvantages are related to claustrophobia and MRI artifacts, which are more prevalent compared with PET/CT (44). Some other relevant deficits are being discussed as follows.

#### Attenuation Correction of PET Data

Attenuation correction is challenging with PET/MRI because MRI cannot directly assess tissue density, particularly for lung and bone tissues. On the first clinical PET/MRI systems, the T1-weighted Dixon MRI sequence was used to segment the MRI data into different tissue classes (e.g., air, lung, soft-tissue, and fat) and to derive the attenuation maps for PET (45). This method provides an acceptable approximation of density for soft-tissue and fat; however, the differentiation of (cortical) bone from air remains challenging as both tissues have near-zero MRI signal intensities resulting in suboptimal PET attenuation corrections. Various other MRI-based attenuation correction methods have been introduced to enhance bone vs. air segmentation, including a combination of Dixon sequence with an ultra-short echo time sequence (45, 46). The most advanced method in the clinical practice to date is based on a precompiled atlas of paired MRI and CT data and an algorithm that generates pseudo-CT images from MRI data. The pseudo-CT data are then converted to PET attenuation maps (47). Despite methodological challenges, MRI-based attenuation correction is no longer an impediment to the clinical adoption of PET/MRI technology (48–51). MRI-based attenuation correction is becoming similar to the CT-based method; however, cautions remain when comparing SUV values between a PET/CT and PET/MRI system for treatment monitoring. Particularly, MRI-based attenuation is still suboptimal for bone tissue and may cause an underestimation of SUV (49).

#### Lung Lesion Detection

CT provides the advantage of high spatial resolution for pulmonary tissue and is considered the reference standard for lung lesion detection. MRI plays only a minor role in this regard because of methodologic and physical shortcomings, mainly attributed to the low proton density in the lungs and respiratory artifacts. Sawicki et al. have shown that the detection and characterization of lung lesions 10 mm or larger are comparable between PET/CT and PET/MRI, but the detection rate for lesions <10 mm is suboptimal with PET/MRI (52). MRI showed an overall detection rate of 66.8%. The detection rate of MRI for lesions <10 mm was 45.9% compared with CT, and lesion size was smaller on MRI (<0.05), which overall represents a risk of missing small pulmonary metastasis on PET/MRI. Despite recent efforts to introduce new MR sequences, the diagnostic accuracy of MRI for lung lesion detection remains inferior to CT (34, 52, 53). In the clinical practice, an unenhanced, breath-hold CT is often recommended in a patient undergoing PET/MRI to rule out small pulmonary metastasis.

#### Radiation Treatment Planning

CT plays a crucial role in radiotherapy planning. Most importantly, there is a close linear correlation between the voxel intensity at CT and the electron density of tissues within image voxels, enabling the attenuation of various tissues to be calculated. MRI has been routinely used to assist with tumor contouring after co-registration with the simulation CT scan. However, a major disadvantage of MRI is that the information on electron density required for radiation treatment planning can only be derived indirectly, not directly (54). Current efforts aiming at MRI-based simulation and treatment planning are encouraging but merely represent feasibility attempts to introduce hybrid PET/MRI into radiotherapy (55, 56). Current PET/MRI protocols optimized for diagnostic imaging may not be appropriate for radiation treatment planning, and special accommodations in hardware and software are required to be able to accomplish MRI-derived radiation treatment planning. At present, PET/CT imaging is the most valuable tool for oncologic patients as it provides direct input to diagnosis and staging as well as radiation treatment planning. The limitations mentioned above represent a major hurdle for the clinical utility of hybrid PET/MRI and help explain why this technology is currently not feasible for a one-stop solution for oncologic patients.

## Conclusion

Current literature supports the notion that F-18 PET/MRI provides greater diagnostic confidence and accuracy than PET/CT in the staging of pelvic malignancies in women. Most importantly, PET/MRI complements the FIGO staging and has the potential to impact clinical decision and treatment strategy. For disease restaging, current data indicate that PET/MRI performs equivalently to PET/CT. There appears to be a mild-moderate inverse correlation between SUV and ADC values, which could be a valuable tool to predict tumor grading and nodal disease as well as distant metastasis. It remains to be seen as to whether multi-parametric PET/MRI imaging could prove valuable for prognostication and outcome. PET/MRI provides the opportunity for reduced radiation exposure, which is particularly relevant for a young female in need of multiple scans for treatment monitoring and follow-up. Fast acquisition protocols and optimized methods for attenuation correction are still evolving. Significant limitations of PET/MRI remains, such as suboptimal detectability of small pulmonary nodules and lack of utility for radiation treatment planning, which pose an impediment in making PET/MRI a viable one-stop-shop imaging option to compete with PET/CT.

## Author Contributions

All authors listed have made a substantial, direct and intellectual contribution to the work, and approved it for publication.

## Conflict of Interest

The authors declare that the research was conducted in the absence of any commercial or financial relationships that could be construed as a potential conflict of interest.
